# Somatic copy number alterations in gastric adenocarcinomas among Asian and Western patients

**DOI:** 10.1371/journal.pone.0176045

**Published:** 2017-04-20

**Authors:** Steven E. Schumacher, Byoung Yong Shim, Giovanni Corso, Min-Hee Ryu, Yoon-Koo Kang, Franco Roviello, Gordon Saksena, Shouyong Peng, Ramesh A. Shivdasani, Adam J. Bass, Rameen Beroukhim

**Affiliations:** 1 Department of Medical Oncology, Dana-Farber Cancer Institute, Boston, Massachusetts, United States of America; 2 Cancer Program, Broad Institute of MIT and Harvard, Cambridge, Massachusetts, United States of America; 3 Department of Human Pathology, University Hospital, Siena, Italy; 4 Department of Oncology, University of Ulsan College of Medicine, Asan Medical Center, Seoul, Korea; 5 Departments of Medicine, Brigham & Women’s Hospital and Harvard Medical School, Boston, Massachusetts, United States of America; University of Michigan, UNITED STATES

## Abstract

Gastric cancer, a leading worldwide cause of cancer mortality, shows high geographic and ethnic variation in incidence rates, which are highest in East Asia. The anatomic locations and clinical behavior also differ by geography, leading to the controversial idea that Eastern and Western forms of the disease are distinct. In view of these differences, we investigated whether gastric cancers from Eastern and Western patients show distinct genomic profiles. We used high-density profiling of somatic copy-number aberrations to analyze the largest collection to date of gastric adenocarcinomas and utilized genotyping data to rigorously annotate ethnic status. The size of this collection allowed us to accurately identify regions of significant copy-number alteration and separately to evaluate tumors arising in Eastern and Western patients. Among molecular subtypes classified by The Cancer Genome Atlas, the frequency of gastric cancers showing chromosomal instability was modestly higher in Western patients. After accounting for this difference, however, gastric cancers arising in Easterners and Westerners have highly similar somatic copy-number patterns. Only one genomic event, focal deletion of the phosphatase gene *PTPRD*, was significantly enriched in Western cases, though also detected in Eastern cases. Thus, despite the different risk factors and clinical features, gastric cancer appears to be a fundamentally similar disease in both populations and the divergent clinical outcomes cannot be ascribed to different underlying structural somatic genetic aberrations.

## Introduction

Each year more than 1 million people are diagnosed with gastric adenocarcinoma, the third leading cause of global cancer-related death [1]. Gastric cancer is more common in the Far East than in most western regions: incidence in East Asia approaches 50 cases per 100,000 people, about 10 times higher than in North America [1]. Other areas of high incidence include Eastern Europe and Andean regions in South America. Epidemiologic features, anatomic distributions, histologic subtypes, and association with *H*. *pylori* infection also differ between Eastern and Western countries [2]. In the West, for example, tumors of the gastric cardia are more common and associated with gastro-esophageal reflux and obesity, whereas tobacco, diet and *H*. *pylori* make proportionally larger contributions toward gastric cancer risk in Asia [3]. Survival of patients with gastric cancer is also superior in Japan and Korea [2, 3]. Some of this effect may reflect mass-screening and early detection, but survival differences persist after adjusting for treatment centers and disease stage [3–5], with a 5-year post-operative survival rates of 61% in Japan and 23% to 28% in Europe and the United States [6, 7]. Different surgical practices, with more extensive lymph node dissection in Asia, may explain some of this difference, but the benefit of this class of surgery is controversial [8–12] and systemic chemotherapy or biologic agents also produce different response and survival rates in Eastern and Western patients. The substantial differences in epidemiology and outcome have stimulated debate whether gastric adenocarcinomas arising in Eastern and Western individuals represent distinct disease entities. If this is true, they would be predicted to carry distinct genomic features.

Molecular characterization of cancers from different parts of the world provides opportunities to address this question, though it is important also to consider the distinct histologic and molecular subtypes of gastric adenocarcinoma. The Lauren pathologic classification distinguishes two principal types; those with diffuse and those with intestinal histology. The diffuse variant is less associated with *H*. *pylori* and may carry a worse prognosis [13]. The intestinal type, the more prevalent form of gastric cancer, arises through a sequence of chronic inflammation, usually related to *H*. *pylori* infection; mucosal atrophy; intestinal metaplasia progressing to dysplasia; and, eventually, invasive cancer [14]. Comprehensive molecular analysis of 295 gastric cancers recently led to a new classification into four distinct subtypes [15]: one variant characterized by Epstein-Barr virus (EBV) infection, one with microsatellite instability (MSI), a highly aneuploid group with chromosomal instability (CIN), and one composed largely of tumors with stable genomes and diffuse histology. However, subgroup analysis in this study identified no clear enrichment in any group of tumors arising in Eastern or Western individuals [15].

Here we compare somatic genomic alterations between gastric cancers of Eastern and Western origin. Recent studies of cancer genomics have found that distinct patterns in somatic copy-number alterations (SCNAs) can be used to discriminate between cancer types [16] and subtypes [17–19]. We focus on patterns of SCNAs across 657 gastric adenocarcinomas, comprising the largest composite set of this disease studied to date. We mapped with improved accuracy the loci that are subject to recurrent gain or loss and determined the incidence of distinct lesions in cancers of Eastern and Western origin. Our copy-number analysis reveals that the two groups of gastric cancer have highly similar genomes, which provides evidence that the different epidemiologic and clinical features typical of Eastern and Western cases do not represent distinct disease entities.

## Materials and methods

This analysis evaluated a composite collection of gastric adenocarcinomas including 581 tumors that have already been previously published and another 76 tumors being first reported in this study. The 76 novel tissue samples selected for this study were provided by the Bio-Resource Center of Asan Medical Center, Korea Biobank Network (2010-6(25)), and their use for cancer research approved by the Asan Medical Center Institutional Review Board. All samples were fresh-frozen after resection. Cells from gastric tumor samples were evaluated by a pathologist for disease presence and tumor content. DNA was extracted using salt precipitation, quantified with Picogreen dye, and hybridized to SNP 6.0 arrays at the Broad Institute according to the instructions provided by the manufacturer (Affymetrix). The data for 76 tumor and 35 normal samples are available at the Gene Expression Omnibus (GEO) under the accession GSE77775.

Probe-level signal intensities from Affymetrix SNP6.CEL files for 657 gastric tumor samples were combined, calibrated, normalized, and segmented in uniform fashion using the Broad Institute SNP6.0 copy number pipeline (S1 Text). The resulting segmented copy number profiles were analyzed to determine significant recurrent SCNAs using GISTIC 2.0 with noise threshold 0.1, focal cutoff 0.5 chromosome arms, and peak confidence window 0.95. The gene-GISTIC algorithm was used to analyze deletions and arm-level peel-off was used to resolve peaks. Genes were associated with a peak if the peak and gene footprint overlapped; a peak overlapping no genes was associated with the nearest gene.

Genotype calls at the SNP6 loci were made using the Birdseed algorithm [20]. These calls were analyzed to determine the genetic ancestry of the samples using the SmartPCA program from the EIGENSTRAT software suite, version 4.2 [21].

Genomic disruption of a sample was measured by the fraction of the genome differing from the median copy number by more than 0.1. Tumor purity and ploidy were determined using the HAPSEG [22] and ABSOLUTE [23] methods for 462 of our 657 samples. Where available, we used these purity/ploidy values to correct a each sample’s copy number profile to remove the effect of admixed normal cells as described previously [24]. SCNAs were called by comparing the corrected profile to a threshold of 0.2 above and below the median.

We used the support vector machine functions from the Matlab Machine Learning Toolbox (release 2012b) to classify our samples into CIN and non-CIN subtypes using a vector space defined by arm-level median copy number and a Gaussian radial basis function kernel with σ = 1.

Focal SCNAs were distinguished from arm-level SCNAs by the ziggurat deconstruction part of the GISTIC 2.0 analysis. Chromosome arm rates were assessed using median purity-corrected copy levels, and significant differences were tested using a Fisher exact test for each arm. To test for significant differences in focal SCNAs we used a permutation test developed to identify correlations that controls for focal event rates and subtype structure [24]. We looked for correlations between East-West cohort membership and focal events within the significant regions identified by our GISTIC analysis by running 49,000 permutations that controlled for CIN status and focal genomic disruption in the ISAR-corrected data. We excluded underpowered loci from the FDR calculation.

Throughout this study, we considered one of multiple hypotheses significant if its false discovery rate (FDR) was < 0.05 [25] and a single test significant if P < 0.05. We compared distributions of values (genomic disruption, purity, event counts) using a two-sided Wilcoxon rank sum test; for categorical comparisons we used a two-sided Fisher exact test.

## Results

### Analysis of somatic copy number profiles

We analyzed copy-number profiles in 657 gastric adenocarcinomas that had all been using Affymetrix SNP 6.0 microarrays. The 76 novel tumor samples presented in this study were entirely from Korean patients. We combined these with 95 cases from an Italian cohort published in a study of gut adenocarcinomas [26], 193 cases from a published Singaporean study [27] and 293 cases published by The Cancer Genome Atlas (TCGA) Research Network [15]. Within the TCGA cohort, 54 cases were from Asian countries, predominantly South Korea and Vietnam, and 239 cases were from Western countries, including Russia and Ukraine. We first used the dataset to define recurrent chromosome arm-level and focal alterations, on the premise that a large combined dataset provides the power to detect rare events and to refine putative gene targets within regions of recurrent alteration. Defining key recurrent alterations across this large tumor set also enables systematic estimation of their prevalence in cases of different ethnic origin. All SNP profiles were uniformly re-analyzed (see Methods) and we identified recurrent events using GISTIC2.0 [16, 28].

**Fig 1:**
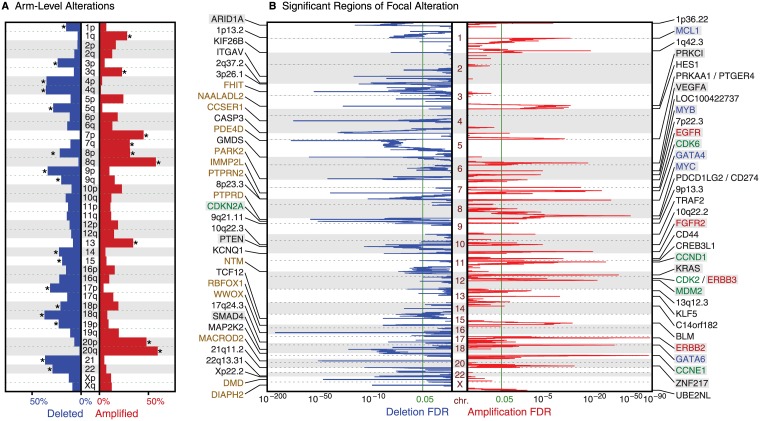
Significant regions of (A) arm-level and (B) focal somatic copy number alteration across the genome (y-axis). The x-axis indicates frequencies (A) or significance (as FDR q-values, B). Arms considered significant (q<0.05) are marked with an asterisk; the significance levels of focal events are shown as green lines. The 35 most significant focal regions of each SCNA type are labeled by associated single genes, putative drivers, or cytoband location. Labels of known oncogenes and tumor suppressors are highlighted in gray; tyrosine kinase genes are red, cell-cycle genes are green, transcription factors are blue text and large genes are brown.

The most significantly recurrent (q<10^−6^) arm-level gains occurred on chromosome arms 20q (59%), 20p (52%), 8q (55%), 8p (42%), 7p (43%), 7q (36%), 13q (37%), and 1q (25%) (Fig 1A). The most significant losses were of 18q (40%), 21q (39%), 9p (37%), 4p (36%), 4q (35%), 17p (32%), 22q (31%), 5q (27%) and 9q (26%). Thus, chromosome 8 showed significant rates of whole-chromosome gain and, among samples without such gain, significant rates of 8p arm-level loss. We also identified 83 regions of significant focal copy-number alteration (FDR<0.05; Fig 1B, S2 Table), including 34 regions of recurrent amplification and 49 regions of significant deletion. For each such area, we focused on the sub-region showing maximal copy-number change, which would be expected to contain the oncogene and tumor suppressor gene targets.

Thirteen amplification peaks contained or were immediately adjacent to a single putative target gene, including eight established oncogenes (*ERBB2*, *CCNE1*, *KRAS*, *FGFR2*, *MYC*, *GATA6*, *ZNF217*, and *VEGFA*). The remaining five peaks contained the stem cell marker *CD44*, a transcription factor (*CREB3L1*) that activates VEGFA expression [29], a regulator of epithelial proliferation (*KLF5*), and long non-coding RNAs *LOC100422737* and *LOC101927851*. Twenty-one amplified areas contained two or more genes. Of these, 8 regions contained previously characterized amplified oncogenes: *GATA4*, *CCND1*, *CDK6*, *MDM2*, *EGFR*, *MCL1*, *ERBB3* and *MYB*; this is the first report of significant and nearly isolated *MYB* amplification in gastric adenocarcinoma (S1A Fig). The *ERBB3* region contains 12 genes, including the cyclin-dependent kinase *CDK2*, which acts with Cyclin E1 to phosphorylate Rb and control entry into S-phase of the cell cycle. The driver genes in the remaining 15 amplified regions are unclear. The 11 genes contained in 5p13.1 include *PRKAA1* and *PTGER4*, which neighbor a gastric cancer risk allele found in genome-wide association studies [30, 31].

Among the 49 deletion peaks, 25 lie in regions that may be mechanistically prone to deletion rather than reflecting positive selective pressure. Seventeen of these peaks contained genes that are among the 100 largest in terms of the length of their footprint across genomic DNA (*WWOX*, *PDE4D*, *CCSER1*, *GRID2*, *PTPRD*, *FHIT*, *DMD*, *PARK2*, *IMMP2L*, *DIAPH2*, *PTPRN2*, *NTM*, *DSCAM*, *PARD3B*, *MGAT4C*, *RBFOX1*, and *NAALADL2*). Deletion of these genes has previously been ascribed to local structural fragility or a local paucity of essential genes [16, 32] [33], though some are also implicated as tumor suppressors [34–36]. Seven other peaks border telomeres, which are mechanistically vulnerable to deletion [24]. Five known tumor suppressor genes (*CDKN2A*, *PTEN*, *ARID1A*, *SMAD4*, and *SMARCA4*) lie among the 21 deletion peaks that contain fewer than 25 genes and are not located at telomeres or contain genes with large footprints. Among these tumor suppressor genes, *SMARCA4* encodes a component of the SWI/SNF chromatin remodeling complex, and significant deletions have not previously been reported in gastric cancer (S1B Fig). The remaining 16 peak regions of deletion may harbor yet unknown tumor suppressors.

To assess how our large data set improves identification of significant regions, we compared these results to the analysis of focal peaks from The Cancer Genome Atlas (TCGA) study on gastric adenocarcinoma [15]. Our analysis revealed 18 additional significant regions, including those containing *MYB* and *SMARCA4*. Among the 68 peaks common to both studies, 37 peaks were smaller than their counterparts in the TCGA study, indicating improved resolution (S2 Fig, S3 Table) and 21 peaks were of the same size; only 10 peaks became wider. A recurrent amplicon at 9p24.1 provides an example of improved resolution. This peak overlapped with *JAK2*, *PDCD1LG2*, *CD274* and seven other genes in the TCGA analysis but was here narrowed to encompass only *PDCD1LG2* and *CD274*, which encode the immunosuppressant proteins and therapeutic targets *PD-L1* and *PD-L2*. An amplification peak at 3q26.2 overlapped with 102 genes in the TCGA study and was reduced to just three genes, including the putative oncogene *PRKCI*.

### Evaluation of ancestry across gastric cancer samples

**Fig 2:**
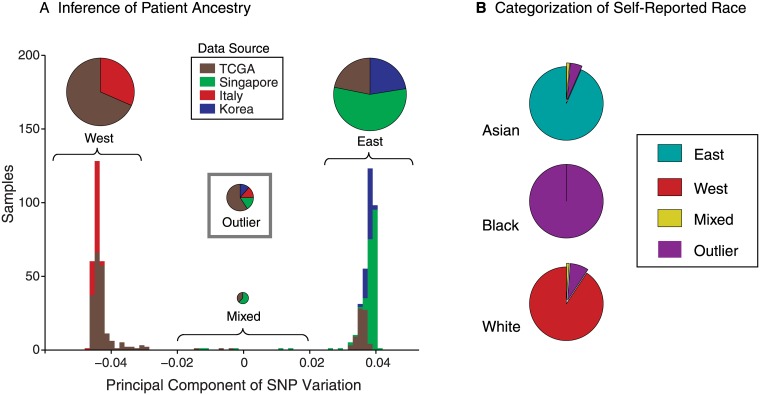
Classification of patient ancestry. (A) Distribution of projections of patient germline SNP genotypes on the principle component of SNP variation. The stacked bars of the histogram are colored by the data source and summarized in overlying proportionately sized pie charts. Outliers do not appear in the histogram. (B) Ancestries determined by genotype among patients reporting Asian, Black, and White ancestry, respectively.

As our collection included large numbers of cases arising in East Asian or Caucasian patients, we could compare somatic genetic alterations in the two populations, provided we could classify ancestry with confidence. To this end, we first applied principal component analysis (PCA) to the germline SNP calls and robustly classified 605 of the 657 samples into two distinct groups by the primary component, indicating two ethnically distinct populations (Fig 2A). This component had an eigenvalue of 50.9, more than ten times stronger than the secondary component’s eigenvalue of 4.7. The remaining 52 samples were unclassified outliers or may reflect mixed ancestry and included most cases arising in African Americans. Notably, all evaluable Korean samples segregated distinctly from the Italian samples and, within the TCGA cohort, the reported ethnic classification was perfectly concordant with how Eastern or Western patients were aggregated in our PCA approach (Fig 2B). Accordingly, we used the primary SNP component to classify the 605 non-ambiguous patients into Eastern and Western cohorts of 323 and 282 patients, respectively (S1 Table).

### Differences in disease subtype between Eastern and Western cohorts

**Fig 3:**
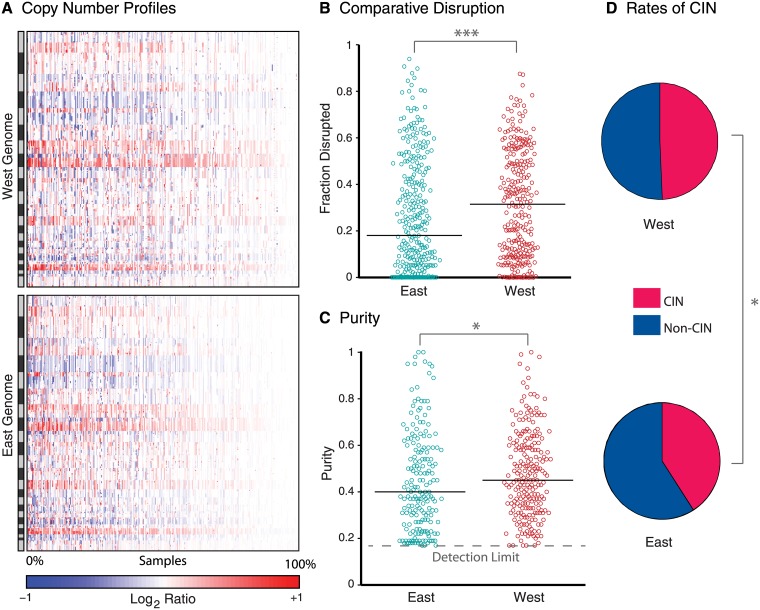
Genomic disruption between Eastern and Western samples. (A) Copy number profiles of Western (top) and Eastern (bottom) samples (x-axis; decreasing genomic disruption towards the left) across the genome (y-axis). Amplifications are in red and deletions in blue. (B) Fraction of the genome disrupted and (C) purity estimates of samples (circles) in each cohort. Solid lines represent median values; the dashed line represents the minimum purity detection limit. (D) Rates of CIN in each cohorts. Single and triple asterisks indicate p≤0.05 and p≤0.001, respectively.

Overall, Western cases exhibited more genomic disruption than Eastern cases (P = 0.0001, Fig 3A and 3B), which could occur for three reasons. First, the Eastern cohort may include more samples with low tumor content, obscuring SCNAs in that population. Second, subtypes of gastric cancer with greater disruption may be genuinely more prevalent in the Western cohort. In particular, the TCGA analysis revealed that gastric cancers with chromosomal instability (CIN) have more frequent copy-number alterations than other groups. Third, tumors of the same subtype may exhibit different rates of genomic disruption between the two populations.

**Fig 4:**
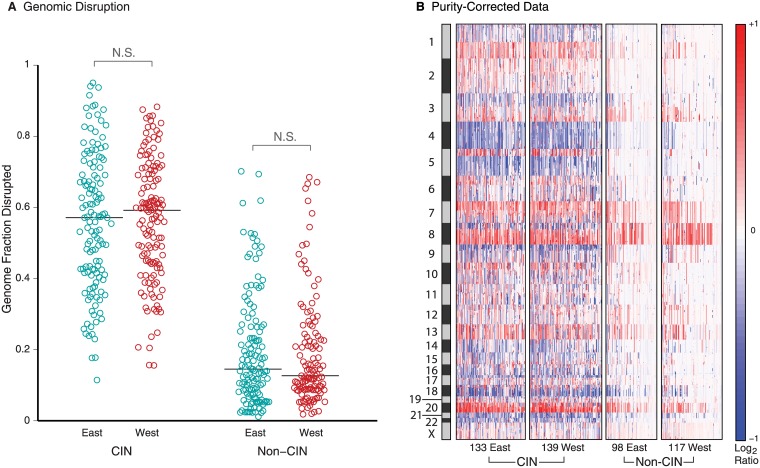
Genomic disruption after purity correction within CIN and non-CIN subtypes. (A) Genomic disruption using purity-corrected data within samples of each subtype. Circles represent samples and lines represent median values. (B) Purity-corrected copy-number profiles arranged by molecular subtype and East/West cohort. Data are presented as in Fig 3A. “N.S.” indicates p>0.05.

Using the ABSOLUTE algorithm [23], we indeed observed higher median tumor purity in the Western than in the Eastern cohort (Fig 3C). The purity of 92 Eastern (28%) and 26 Western tumors (9%) appeared insufficient to make confident SCNA calls, so we excluded these cases from further analysis of ethnic differences. We re-normalized the copy-number profiles of the remaining tumors to remove effects of tumor impurity, using an in-silico admixture removal (ISAR) calculation [23]. After this adjustment, CIN tumors were still significantly more common in the Western than in the Eastern cases (59% vs. 51%, p = 0.024, Fig 3D). We classified CIN across both cohorts by training a support vector machine on the TCGA dataset, where CIN and non-CIN subtypes were known. Within the resulting CIN and non-CIN groups, Eastern and Western cases showed similar levels of overall genome disruption (Fig 4A and 4B). Thus, the observed differences in overall levels of genome disruption reflect differences in tumor purity and modestly different rates of CIN rather than variable rates of genome disruption within CIN and non-CIN groups (Fig 4A).

Surprisingly, the CIN status of our tumors did not correlate significantly with the histological tumor stage, but showed a weak correlation with the intestinal (versus diffuse) Lauren classification (P = 0.04, Fisher exact test). Neither the tumor stage nor its Lauren classification was significantly correlated with East/West status.

We tested the SCNA events called on the purity corrected copy number data for patient group associations using Fisher’s exact test. Unsurprisingly, focal events in most of our regions (61 of 83) were significantly correlated with CIN status, including MYB amplification. SMARCA4 deletion was not significantly associated with CIN. No events were significantly associated with the patient’s Lauren status. However, MYB amplification was significantly associated with tumor stage (FDR = 0.03).

### Genome features in comparable groups of Eastern and Western gastric cancer

Against this backdrop, we identified few differences in specific SCNAs in Eastern and Western cases of gastric cancer. First examining rates of amplification and deletion separately across the two cohorts, we detected larger numbers of focal deletions in the West cohort and of arm-level deletions in the East cohort (P = 0.02 and 0.03, Wilcoxon rank sum test). There were no significant differences in the rates of arm-level or focal amplification events. (S3A Fig). Even after controlling for CIN status, focal deletion rates remained slightly higher in the West cohort in both CIN and non-CIN groups (Wilcoxon P = 0.1 and 0.2 respectively, S3B and S3C Fig), whereas arm-level deletions were enriched among Eastern non-CIN samples (Wilcoxon P = 0.007). This difference was driven by a higher frequency (28%) of Western samples with no arm-level deletions compared to 17% of Eastern cases (S3D Fig). If these samples are excluded, the remaining non-CIN samples exhibited no significant difference in arm-level deletion rates, but retained a significant difference in focal deletion rates (P = 0.007, S3E Fig).

We further explored the decreased rates of arm-level deletions in Western non-CIN samples. Among TCGA samples, lack of arm-level deletions was significantly correlated with the MSI subtype after controlling for CIN (P = 10^−7^, S3F Fig). An independent assessment of MSI was available in the TCGA samples but not the other cohorts. These results suggest that the decreased arm-level deletion rates among Western samples could be due to a higher rate of MSI. Within the TCGA cohort, a slightly higher fraction of Western samples exhibited MSI relative to Eastern samples (23% vs 21%), but the difference was not statistically significant (p = 0.9).

**Fig 5:**
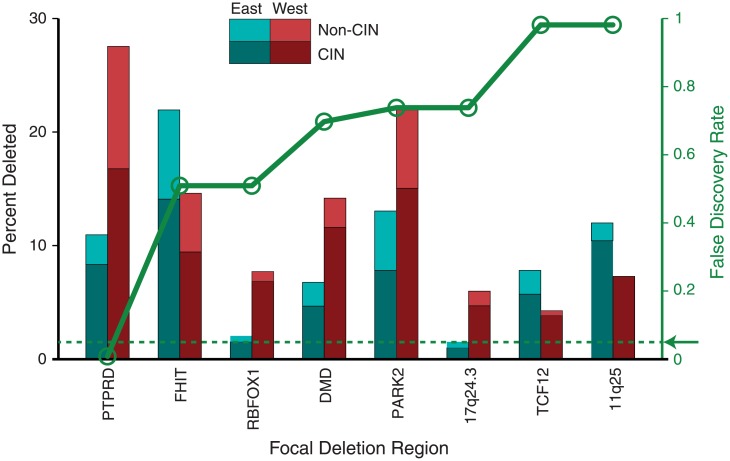
Event frequencies at regions of significant focal SCNA. A bar chart of comparative event frequencies (black scale) is overlaid with a plot of the significance of the event rate difference (green scale). Only those with FDR q<1.0 (all deletions) are shown. The arrow and dashed line indicate the significance cutoff of 0.05.

We next evaluated differences between rates of individual arm-level and focal SCNAs between Eastern and Western samples. To this end, we used a permutation test that controls for both overall levels of genomic disruption and disease subtype (CIN vs non-CIN; Fig 5; S4 and S5 Tables; and S4 Fig) [24]. Although individual chromosome arms exhibited different rates of gain and loss (S4 Fig), none of these reached statistical significance (S5A–S5C Table). We then compared rates of individual focal SCNAs at all significant peak regions of alteration between Eastern and Western samples, using a permutation test that controls for both overall levels of genomic disruption and disease subtype (CIN vs non-CIN) [24].

Only focal deletion of the phosphatase gene *PTPRD* reached significance (FDR = 0.007; Fig 5, S5 Fig, S4A–S4C Table), occurring in 27% of Western and 11% of Eastern samples for a combined rate of 20. This difference in rates was evident in both CIN (West 29%, East 13%) and non-CIN (West 25%, East 7%) cases. Varying the permutation test to control for tumor stage or histological subtype instead of CIN also found PTPRD deletion as the event most correlated with East/West status (S4D and S4E Table). *PTPRD* deletions were enriched among Western patients (P = 0.004) even within the TCGA cohort, suggesting that the difference was not due to experimental technique. Among TCGA cases, where clinical and pathology information is the most thorough, *PTPRD* deletions did not cluster in a specific location, gender, Lauren or molecular subtype of gastric cancer. To ensure that our analysis was not missing the correlation of a region found to be significant in only East or West, we repeated the CIN-controlled analysis adding all significant regions found in only one cohort. Again, only PTPRD deletions were found to be significantly correlated (S4F Table).

## Discussion

There has been substantial debate within the gastric cancer field about whether there exist intrinsic biologic differences between gastric cancers in patients from the Eastern and Western worlds. Our assembly of large genomic SCNA datasets from Eastern and Western gastric cancer patients allowed us to evaluate copy-number differences in somatic genomes of tumors from Eastern and Western populations, groups which show highly divergent incidence and survival rates for gastric cancer. Moreover, the power of this dataset enabled us to refine recurrent copy-number alterations and to newly identify, for example, *MYB* amplifications and *SMARCA4* deletions in this disease. Through our analysis, we detected increased rates of genome disruption in Western cases, with specific increases in focal deletions, especially those involving *PTPRD*, and a relative paucity of arm-level chromosome losses.

The initially observed overall rates of genome disruption largely reflect a combination of variations in tumor purity and modestly different rates of the CIN phenotypes in the Eastern and Western patients in our cohort, rather than divergent frequencies of particular events in Eastern and Western cancers of the same subtype. It is also possible that the decreased rates of arm-level deletions among the Western non-CIN samples in our cohort is due to a modestly higher fraction of MSI+ cancers. Our findings of higher rates of CIN tumors in patients of Western descent is consistent with data demonstrating that CIN tumors are more prevalent in the proximal stomach [15] as a predilection for proximal tumors are a characteristic of Western stomach cancers. Prior studies have documented significantly lower rates of proximal cancers in Asians, including those who have immigrated to the West [37, 38].

Our results must also be evaluated in the context of known differences in clinical practice in the Eastern compared to Western world. In the East, for example, greater surveillance and awareness of gastric cancer, contributes to disease being found at earlier stages [39]. We cannot exclude that the enhanced detection and resection of smaller tumors does not contribute to the lower tumor purity we detected in the Eastern cohort. Additionally, the different rates of distinct biologic subtypes also may contribute to these purity differences. EBV-positive and MSI tumors are both more common in the more distal regions of the stomach. As these tumors have greater inflammatory infiltrates, the presence of such non-malignant cells would lead to reduced tumor cell purity.

A potential enrichment of CIN tumors in Western patients may therefore provide some explanation for why gastric cancer is associated with poorer survival in the West Both genome disruption [40] and cancer of the proximal stomach [41] are associated with poor survival. Indeed, our analysis suggests that the longstanding debate regarding Eastern and Western stomach cancer is confounded by different distributions of stomach cancer subtypes in these populations. After controlling for CIN, gastric cancers arising in Eastern and Western patients showed strikingly similar genomes.

Nevertheless, increased rates of focal deletion, particularly of *PTPRD*, among Western non-CIN samples are not easily explained by varied representations of gastric cancer subtypes. Intriguingly, deletions that we find enriched in the Western patients are not necessarily at loci clearly established to functionally promote tumorigenesis. Even *PTPRD* deletions have been proposed to be secondary to DNA fragility rather than driver events, as *PTPRD* is a large gene and a known fragile site [16, 42]. Our findings raise the additional hypothesis that differences in Eastern and Western germ line haplotypes or environmental exposures generate influence the phenotype of genomic instability leading to alternative rates of alteration of *PTPRD* and other loci.

If CIN tumors are more common in the West, then other subtypes of stomach cancer may be enriched in Eastern populations. One small study did report significant enrichment of MSI+ cases in Japan, compared to the West [43]. Additionally, both MSI+ and EBV+ tumors are less prevalent in the proximal stomach [44, 45] and associated with higher survival [44–46], thus potentially contributing to discrepant survival in Eastern and Western patients. Although gastric cancers with diffuse histology include both those with and without CIN, the greater proportion in the recent TCGA study lacked CIN [15]. While several studies identify higher rates of diffuse-type tumors in Eastern populations [38, 47], other reports note higher rates of diffuse disease in Western patients [39]. Unlike MSI+ and EBV+ tumors, however, diffuse-type gastric cancers carry a worse prognosis, implying that this bias likely contributes little to the survival advantage reported in the East.

Our comparison between populations relied on a strictly genetic designation of ethnicity. As these genetic features overlap with environmental risk factors for gastric cancer, we cannot determine if particular discrepant somatic features of Eastern and Western gastric cancer have a genetic or environmental basis. For example, *H*. *pylori* infection is less prevalent in the West [48] and absence of *H*. *pylori* infection is associated with proximal cancers [49]. Most specimen collections are incompletely annotated for *H*. *pylori* infection because the bacteria is only present in regions of pre-neoplastic gastritis and is typically lost following development of intestinal metaplasia. Therefore, we are not able to specifically query whether *H*. *Pylori* status influences our results. In addition to this limitation, our composite study lacks the complete tumor EBV, MSI and histologic status necessary to completely address these questions, as well as certain basic clinical parameters such as gender, age, and disease treatment.

Within these limitations, our study indicates that gastric adenocarcinoma encompasses distinct biological but not absolute distinct ethnic subtypes. Nevertheless, different ethnic groups may differ in the predisposition to distinct subtypes of gastric cancer for genetic or environmental reasons, and we show that variation in the subtype prevalence accounts for nearly all the difference in the rates of somatic copy-number aberrations. We note that our analysis did not consider differences in gene expression, DNA methylation or gene mutation. Another recent meta-analysis of Eastern and Western stomach cancer patients identified specific inflammatory genes to be enriched in expression in the Western patients [50]. Indeed, further studies of potential differences in the inflammatory composition of tumors of distinct geographic origin are called for and could identify non-genetic differences between tumors which could influence survival and optimal therapy, especially given the burgeoning field of immunotherapy.

Overall, our data support the supposition that Eastern and Western gastric cancers are not fundamentally distinct diseases and are consistent with emerging thinking that rather than geography or ethnicity, it is the molecular subtypes of this disease that are the primary categories we should evaluate to sub-divide these tumors. As we further explore the biology and therapeutics for these cancers in different patient populations, it will be essential to take these molecular subtypes into account to avoid comparisons that are confounded because of distinct distributions of gastric cancer subtypes across different populations of patients.

## Supporting information

S1 Text The SNP 6.0 copy number pipeline. (DOCX)

S1 Fig Focal copy number alterations contributing to novel gastric cancer peaks. Amplified segments are marked red and deleted segments blue across the genome (X-axis) and selected patients (Y-axis). Peak regions are delimited by vertical gray liness. Genes in or near the peak are placed above the heat map with the presumed driver highlighted in green. Shown are (A) the significantly amplified region containing *MYB* on chromosome 6 and (B) the significantly deleted region containing *SMARCA4* on chromosome 19.
(PDF)

S2 Fig Resolution (A) and significance levels (B) of peak regions of focal SCNA in this study (x-axis) and TCGA samples (y-axis), using identical analysis parameters. Matched peaks are shown as circles; unmatched peaks are shown as Xs placed in the margins. Amplifications are in red and deletions are in blue.
(PDF)

S3 Fig Distributions of arm-level (x-axis) and focal (y-axis) event frequencies across samples. Each sample is represented by a circle; colors indicate East/West status or molecular subtypes as indicated). Colored crosses indicate group medians at intersections and group quartiles by extents. Asterisks indicate p<0.05; “N.S.” indicates p>0.05. (A) Gain/amplification and (B) loss/deletion frequencies across all samples. C-D) Loss/deletion frequencies within (C) CIN subtype, (D) non-CIN subtype. (E) Loss/deletion frequencies excluding samples without arm-level deletions. (F) Loss/deletion frequencies across subtypes within TCGA.
(PDF)

S4 Fig Frequencies (x-axis) of arm-level gains (red) and losses (blue) between East (left) and West (right) cohorts. Events are indicated by chromosome arm. Vertical and horizontal arrows summarize the overall and difference in frequencies respectively using an average weighted by arm size. Each panel analyzes a different subgroup with a pie graph indicating the size and East-West composition of each group. (A) All ABSOLUTE-called data. (B) CIN samples. (C) Non-CIN samples. (D) Non-CIN samples with arm-level deletions. (E) Non-CIN samples without arm-level deletions (enriched for MSI). (F) If samples without arm-level deletions are excluded, there are no significant arm-level East-West differences among the remaining samples, although there are still significantly more focal deletions in the West cohort.
(PDF)

S5 Fig IGV view of copy-number profiles at the PTPRD locus among Western (top) and Eastern (bottom) samples. (PDF)

S1 Table Patient samples and characteristics. (XLSX)

S2 Table Peak regions of focal copy number alteration. (A) Peak amplification regions; (B) peak deletion regions; (C) notes legend.
(XLSX)

S3 Table Comparison of TCGA peaks with those of our larger study. (XLSX)

S4 Table East-West focal correlation test results. (A) All samples; (B) samples classified as CIN; (C) samples classified as non-CIN; (D) controlled by tumor stage; (E) controlled by Lauren classification; (F) including peak regions unique to East or West.
(XLSX)

S5 Table East-West arm level comparison. (A) All 423 samples with ABSOLUTE calls; (B) 20 CIN samples; (C) 173 non-CIN samples; (D) 76 non-CIN with arm-level deletions; (E) 97 non-CIN samples without arm-level deletions.
(XLSX)
