# Supplementary material for: Somatic copy number alterations in gastric adenocarcinomas among Asian and Western patients
Source: PLoS One. 2017 Apr 20;12(4):e0176045. doi: 10.1371/journal.pone.0176045 (PMC5398631; doi:10.1371/journal.pone.0176045)
Supplement: S1 Text — (DOCX) [file pone.0176045.s001.docx]

# The SNP 6.0 copy number pipeline

The Affymetrix SNP 6.0 data used in this study was generated from several centers, each likely to have its own approaches to sample preparation, DNA extraction, and array hybridization. To mitigate both data center and batch effects, the raw light intensity data from the original Affymetrix CEL files were uniformly reprocessed using the Broad Institute copy number inference pipeline[1]. This data processing pipeline has been used to process SNP 6.0 data for TCGA solid tumors since 2011[2-17].

The first stage of the pipeline, SNPFileCreator, normalizes the signal intensities across samples and probes using a variant of the dChip signal intensity processing algorithm[18]. The purpose of this step is to uniformly rescale the intensity of signal for each array experiment. Following SNPFileCreator, signal intensity data are calibrated to copy number using the Birdseed algorithm[19]. In brief, Birdseed uses the germline variation in copy number measured at the SNP probes in the array to determine the average behavior of each probe for the batch. The non-SNP probes in the array are calibrated using their sequence similarity to X chromosome probes on which dosage experiments have been performed using cell lines with known copies of X. After this step, each probe on the array has an average calibrated copy number across each batch.

The next step in the pipeline, tangent normalization, is a linear method that removes correlated noise from individual sample data by representing each sample in a high dimensional space by projecting each tumor onto the kernel of the subspace spanned by a large number of normals. This approach to normalization is in direct contrast to the traditional method of comparing a tumor to its matched normal. The underlying principle is that the best match to the systematic noise found in a given tumor samples comes not from any single normal sample, but rather from a linear combination of non-tumor germline DNA samples from a panel representing a very large number of experimental conditions. For our study, we used a first stage combination derived from a panel of 3400 The Cancer Genome Atlas (TCGA) non-tumor DNA samples. Subsequently, we utilized a smaller second stage panel of non-tumor DNA samples from the same batch/center as the tumor being normalized. The noise in the copy number signal is predominantly multiplicative; therefore the data are log-transformed prior to tangent normalization. Extreme outliers are removed prior to normalization to ensure robust results from this linear method.

The final step in the copy number pipeline is averaging the signal from contiguous probes into segments, for which we use the Circular Binary Segmentation (CBS) algorithm [20]. CBS models the probe level data as contiguous segments of constant copy number separated by breakpoints. The copy number pipeline applies quality control metrics to the data at two steps: signal/noise ratio is determined after Birdseed, and the number of segments is determined after CBS. Samples with outlier values for these metrics were excluded from our study.

A fundamental problem in the copy number analysis of cancer is distinguishing somatic events present in tumor samples from germ line copy number variations (CNVs) that are also present in the matched normal sample. Consistent with our pooled approach, we analyze the aggregate of normal samples after the CBS step and exclude regions that exhibit a significant amount of variation from downstream analysis. The excluded CNV regions represent a combination of germ line events and platform-specific artifacts that would otherwise confound downstream analyses.

## References

1. Tabak B, Saksena G, Monti S, Gentry J, Hernandez BC, O'Kelly M, et al. The Tangent copy-number inference pipeline for cancer genome analyses: (in submission); 2016. Available from: <http://www.broadinstitute.org/cancer/cga/copynumber_pipeline>.

2. The Cancer Genome Atlas Research Network. Comprehensive molecular portraits of human breast tumours. Nature. 2012;490(7418):61-70.

3. The Cancer Genome Atlas Research Network. Comprehensive genomic characterization of squamous cell lung cancers. Nature. 2012;489(7417):519-25.

4. The Cancer Genome Atlas Research Network. Integrated genomic characterization of endometrial carcinoma. Nature. 2013;497(7447):67-73.

5. The Cancer Genome Atlas Research Network. Comprehensive molecular characterization of clear cell renal cell carcinoma. Nature. 2013;499(7456):43-9.

6. Brennan Cameron W, Verhaak Roel GW, McKenna A, Campos B, Noushmehr H, Salama Sofie R, et al. The Somatic Genomic Landscape of Glioblastoma. Cell. 155(2):462-77. doi: 10.1016/j.cell.2013.09.034.

7. The Cancer Genome Atlas Research Network. Comprehensive molecular characterization of urothelial bladder carcinoma. Nature. 2014;507(7492):315-22.

8. The Cancer Genome Atlas Research Network. Comprehensive molecular profiling of lung adenocarcinoma. Nature. 2014;511(7511):543-50.

9. The Cancer Genome Atlas Research Network. Comprehensive molecular characterization of gastric adenocarcinoma. Nature. 2014;513(7517):202-9.

10. Davis Caleb F, Ricketts CJ, Wang M, Yang L, Cherniack Andrew D, Shen H, et al. The Somatic Genomic Landscape of Chromophobe Renal Cell Carcinoma. Cancer Cell. 26(3):319-30. doi: 10.1016/j.ccr.2014.07.014.

11. Agrawal N, Akbani R, Aksoy BA, Ally A, Arachchi H, Asa Sylvia L, et al. Integrated Genomic Characterization of Papillary Thyroid Carcinoma. Cell. 159(3):676-90. doi: 10.1016/j.cell.2014.09.050.

12. The Cancer Genome Atlas Network. Comprehensive genomic characterization of head and neck squamous cell carcinomas. Nature. 2015;517(7536):576-82.

13. The Cancer Genome Atlas Research Network. Comprehensive, Integrative Genomic Analysis of Diffuse Lower-Grade Gliomas. New England Journal of Medicine. 2015;372(26):2481-98. doi: doi:10.1056/NEJMoa1402121. PubMed PMID: 26061751.

14. Akbani R, Akdemir Kadir C, Aksoy BA, Albert M, Ally A, Amin Samirkumar B, et al. Genomic Classification of Cutaneous Melanoma. Cell. 161(7):1681-96. doi: 10.1016/j.cell.2015.05.044.

15. Ciriello G, Gatza Michael L, Beck Andrew H, Wilkerson Matthew D, Rhie Suhn K, Pastore A, et al. Comprehensive Molecular Portraits of Invasive Lobular Breast Cancer. Cell. 163(2):506-19. doi: 10.1016/j.cell.2015.09.033.

16. The Cancer Genome Atlas Research Network. Comprehensive Molecular Characterization of Papillary Renal-Cell Carcinoma. New England Journal of Medicine. 2016;374(2):135-45. doi: doi:10.1056/NEJMoa1505917. PubMed PMID: 26536169.

17. Abeshouse A, Ahn J, Akbani R, Ally A, Amin S, Andry Christopher D, et al. The Molecular Taxonomy of Primary Prostate Cancer. Cell. 163(4):1011-25. doi: 10.1016/j.cell.2015.10.025.

18. Li C, Wong WH. Model-based analysis of oligonucleotide arrays: expression index computation and outlier detection. Proceedings of the National Academy of Sciences of the United States of America. 2001;98(1):31-6. Epub 2001/01/03. doi: 10.1073/pnas.011404098. PubMed PMID: 11134512; PubMed Central PMCID: PMCPMC14539.

19. Korn JM, Kuruvilla FG, McCarroll SA, Wysoker A, Nemesh J, Cawley S, et al. Integrated genotype calling and association analysis of SNPs, common copy number polymorphisms and rare CNVs. Nature genetics. 2008;40(10):1253-60. Epub 2008/09/09. doi: 10.1038/ng.237. PubMed PMID: 18776909; PubMed Central PMCID: PMCPMC2756534.

20. Venkatraman ES, Olshen AB. A faster circular binary segmentation algorithm for the analysis of array CGH data. Bioinformatics (Oxford, England). 2007;23(6):657-63. Epub 2007/01/20. doi: 10.1093/bioinformatics/btl646. PubMed PMID: 17234643.
