# Supplementary material for: Somatic copy number alterations in gastric adenocarcinomas among Asian and Western patients
Source: PLoS One. 2017 Apr 20;12(4):e0176045. doi: 10.1371/journal.pone.0176045 (PMC5398631; doi:10.1371/journal.pone.0176045)

S1 Fig. Copy number alterations contributing to novel gastric cancer peaks. Amplified segments are marked red and deleted segments blue across the genome (X-axis) and selected patients (Y-axis). Peak regions are delimited by vertical gray liness. Genes in or near the peak are placed above the heat map with the presumed driver highlighted in green. Shown are (A) the significantly amplified region containing *MYB* on chromosome 6 and (B) the significantly deleted region containing *SMARCA4* on chromosome 19.

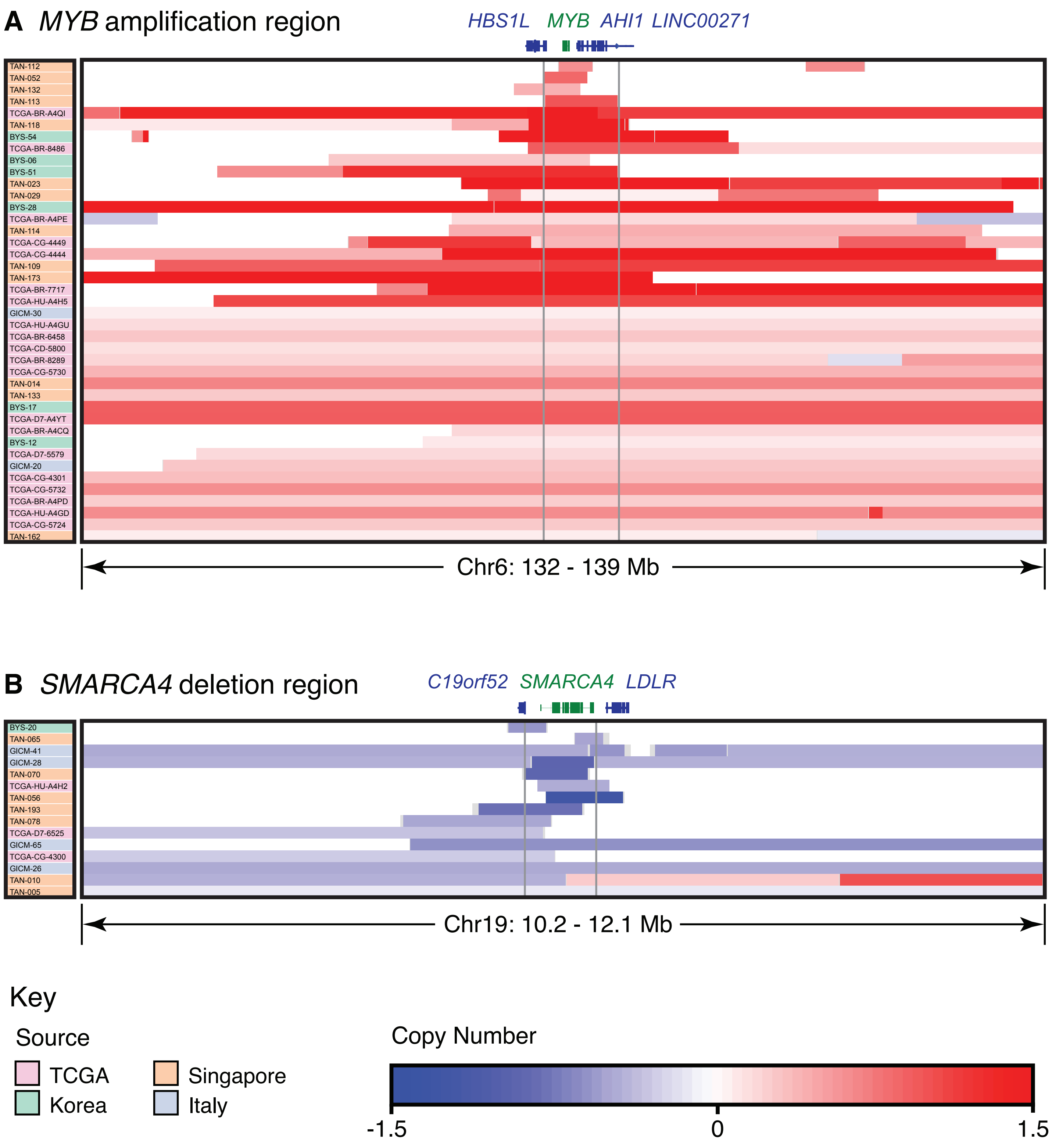

Supplement: S1 Fig — Amplified segments are marked red and deleted segments blue across the genome (X-axis) and selected patients (Y-axis). Peak regions are delimited by vertical gray liness. Genes in or near the peak are placed above the heat map with the presumed driver highlighted in green. Shown are (A) the significantly amplified region containing MYB on chromosome 6 and (B) the significantly deleted region containing SMARCA4 on chromosome 19. (PDF) [file pone.0176045.s002.pdf]
