# Supplementary material for: Somatic copy number alterations in gastric adenocarcinomas among Asian and Western patients
Source: PLoS One. 2017 Apr 20;12(4):e0176045. doi: 10.1371/journal.pone.0176045 (PMC5398631; doi:10.1371/journal.pone.0176045)

S2 Fig. Resolution (A) and significance levels (B) of peak regions focal SCNA in this study (x-axis) and TCGA samples (y-axis), using identical analysis parameters. Matched peaks are shown as circles; unmatched peaks are shown as Xs placed in the margins. Amplifications are in red and deletions are in blue.

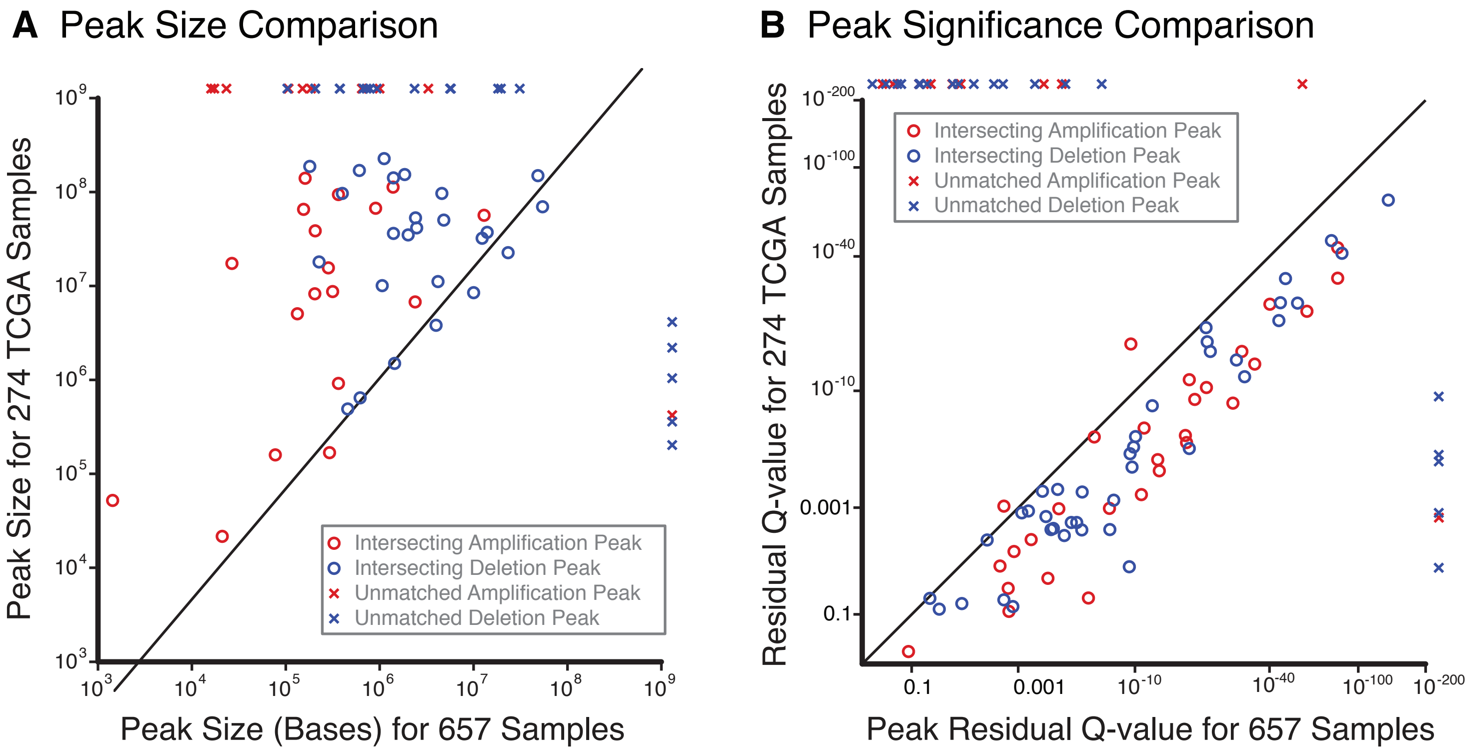

Supplement: S2 Fig — Matched peaks are shown as circles; unmatched peaks are shown as Xs placed in the margins. Amplifications are in red and deletions are in blue. (PDF) [file pone.0176045.s003.pdf]
