# Supplementary material for: Somatic copy number alterations in gastric adenocarcinomas among Asian and Western patients
Source: PLoS One. 2017 Apr 20;12(4):e0176045. doi: 10.1371/journal.pone.0176045 (PMC5398631; doi:10.1371/journal.pone.0176045)

S3 Fig. Distributions of arm-level (x-axis) and focal (y-axis) event frequencies across samples. Each sample is represented by a circle; colors indicate East/West status or molecular subtypes as indicated). Colored crosses indicate group medians at intersections and group quartiles by extents. Asterisks indicate $p < 0.05$; "N.S." indicates $p > 0.05$. A) Gain/amplification and (B) loss/deletion freq-uencies (across all samples. (C-D) Loss/deletion frequencies within (C) CIN subtype, (D) non-CIN subtype. (E) Loss/deletion frequencies excluding samples without arm-level deletions. (F) Loss/deletion frequencies across subtypes within TCGA.

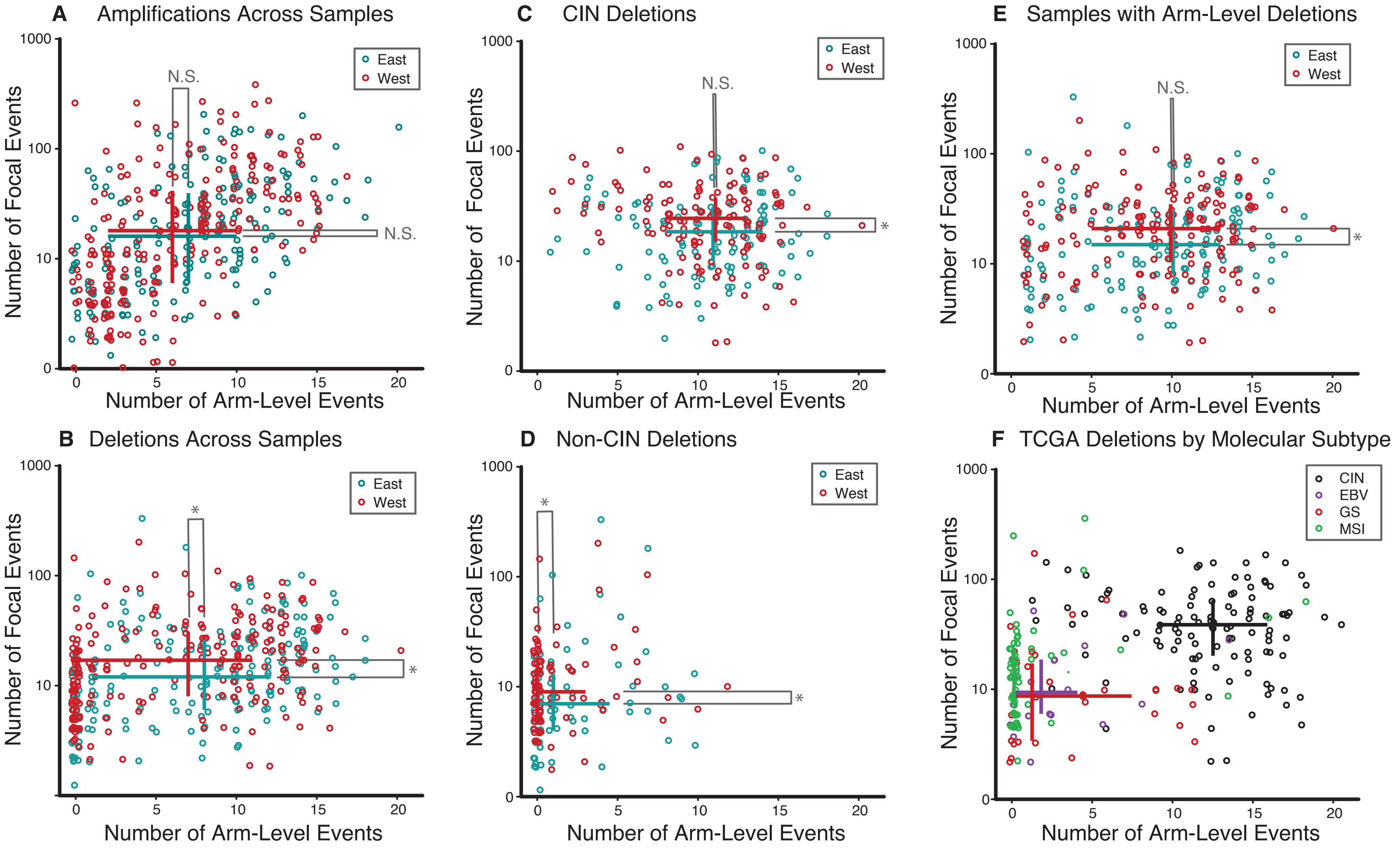

Supplement: S3 Fig — Each sample is represented by a circle; colors indicate East/West status or molecular subtypes as indicated). Colored crosses indicate group medians at intersections and group quartiles by extents. Asterisks indicate p<0.05; “N.S.” indicates p>0.05. (A) Gain/amplification and (B) loss/deletion frequencies across all samples. C-D) Loss/deletion frequencies within (C) CIN subtype, (D) non-CIN subtype. (E) Loss/deletion frequencies excluding samples without arm-level deletions. (F) Loss/deletion frequencies across subtypes within TCGA. (PDF) [file pone.0176045.s004.pdf]
