# Supplementary material for: Somatic copy number alterations in gastric adenocarcinomas among Asian and Western patients
Source: PLoS One. 2017 Apr 20;12(4):e0176045. doi: 10.1371/journal.pone.0176045 (PMC5398631; doi:10.1371/journal.pone.0176045)

S4 Fig. Frequencies (x-axis) of arm-level gains (red) and losses (blue) between East (left) and West (right) cohorts. Events are indicated by chromosome arm. Vertical and horizontal arrows summarize the overall and difference in frequencies respectively using an average weighted by arm size. Each panel analyzes a different subgroup with a pie graph indicating the size of the group and its East-West composition. (A) All ABSOLUTE-called data. (B) CIN samples. (C) Non-CIN samples. (D) Non-CIN samples with arm-level deletions. (E) Non-CIN samples without arm-level deletions (enriched for MSI).

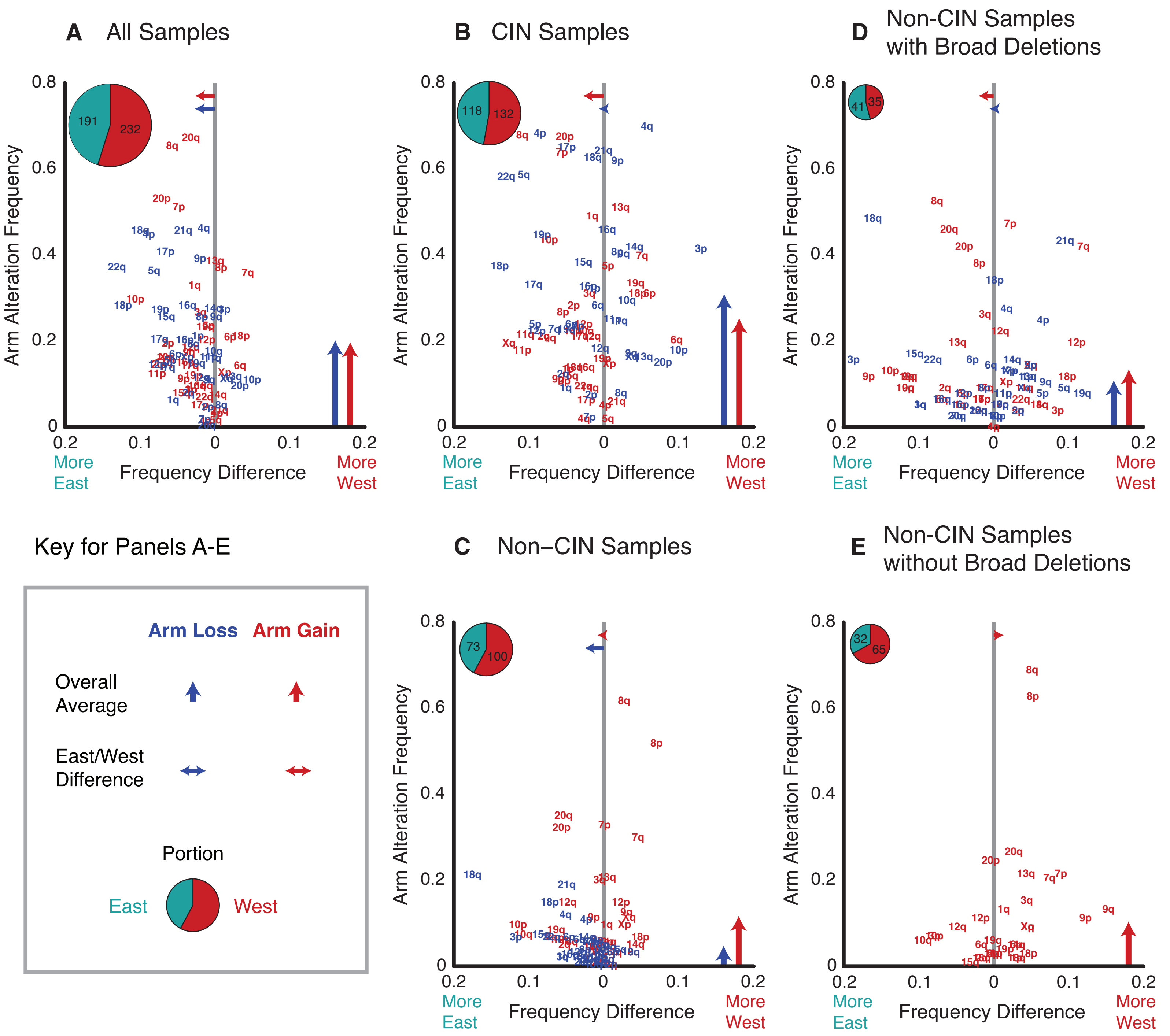

Supplement: S4 Fig — Events are indicated by chromosome arm. Vertical and horizontal arrows summarize the overall and difference in frequencies respectively using an average weighted by arm size. Each panel analyzes a different subgroup with a pie graph indicating the size and East-West composition of each group. (A) All ABSOLUTE-called data. (B) CIN samples. (C) Non-CIN samples. (D) Non-CIN samples with arm-level deletions. (E) Non-CIN samples without arm-level deletions (enriched for MSI). (F) If samples without arm-level deletions are excluded, there are no significant arm-level East-West differences among the remaining samples, although there are still significantly more focal deletions in the West cohort. (PDF) [file pone.0176045.s005.pdf]
