# Supplementary material for: Somatic copy number alterations in gastric adenocarcinomas among Asian and Western patients
Source: PLoS One. 2017 Apr 20;12(4):e0176045. doi: 10.1371/journal.pone.0176045 (PMC5398631; doi:10.1371/journal.pone.0176045)

Figure S5. Focal copy-number profiles at the *PTPRD* locus across all Western (top) and Eastern (bottom) samples.

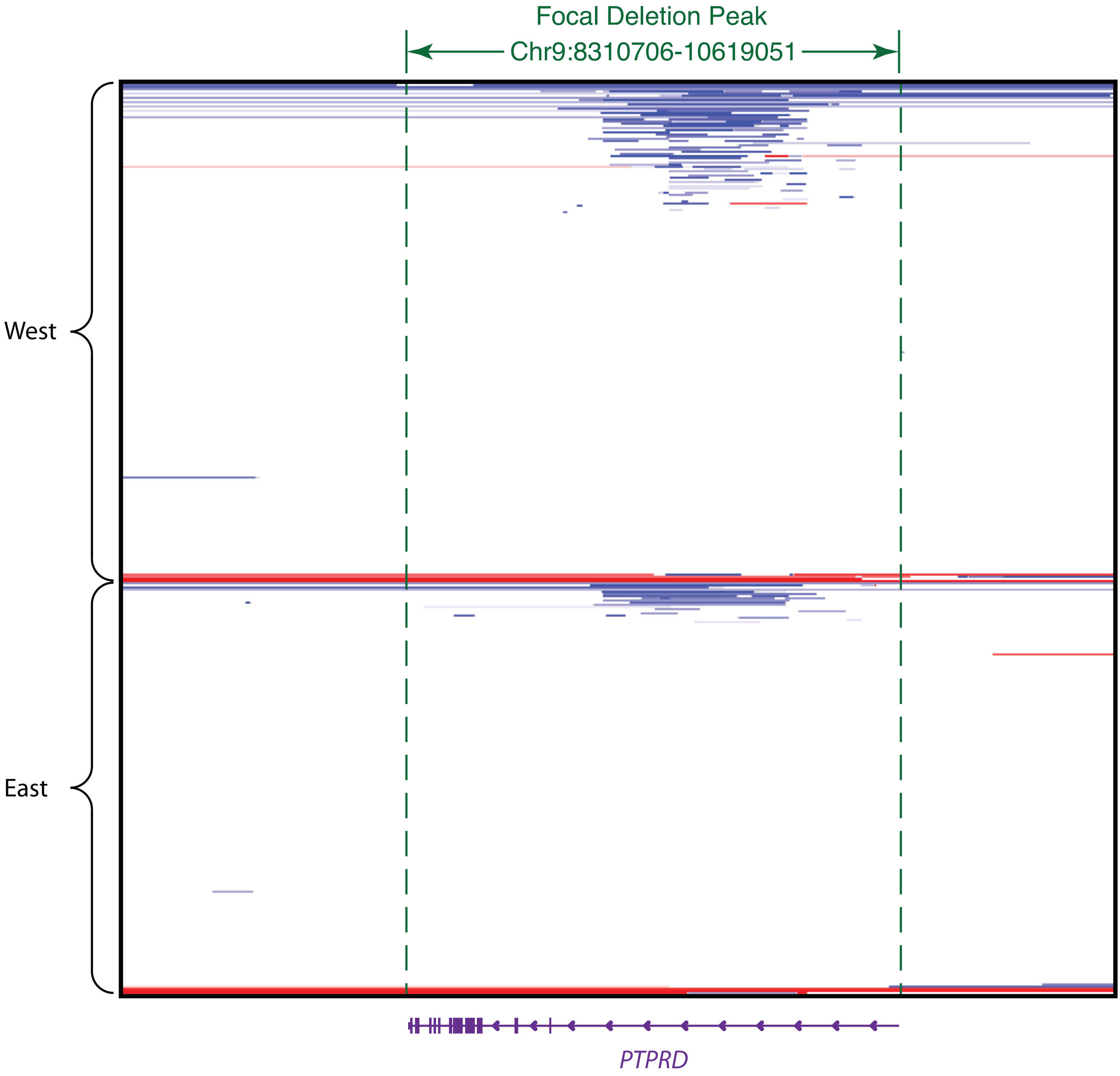

Supplement: S5 Fig — (PDF) [file pone.0176045.s006.pdf]
